# Clinical and Laboratory Characteristics of Hyperprolactinemia in Children and Adolescents: National Survey

**DOI:** 10.4274/jcrpe.galenos.2018.2018.0206

**Published:** 2019-05-28

**Authors:** Erdal Eren, Ayça Törel Ergür, Şükriye Pınar İşgüven, Eda Çelebi Bitkin, Merih Berberoğlu, Zeynep Şıklar, Firdevs Baş, Servet Yel, Serpil Baş, Elif Söbü, Abdullah Bereket, Serap Turan, Halil Sağlam, Zeynep Atay, Oya Ercan, Tülay Güran, Mehmet Emre Atabek, Hüseyin Anıl Korkmaz, Aylin Kılınç Uğurlu, Ayşehan Akıncı, Esra Döğer, Enver Şimşek, Emine Demet Akbaş, Ayhan Abacı, Ülkü Gül, Sezer Acar, Eda Mengen Uçaktürk, Melek Yıldız, Edip Ünal, Ömer Tarım

**Affiliations:** 1 Uludağ University Faculty of Medicine, Department of Pediatric Endocrinology, Bursa, Turkey; 2 Kırıkkale University Faculty of Medicine, Department of Pediatric Endocrinology, Kırıkkale, Turkey; 3 Sakarya University Faculty of Medicine, Department of Pediatric Endocrinology, Sakarya, Turkey; 4 Ondokuz Mayıs University Faculty of Medicine, Department of Pediatric Endocrinology, Samsun, Turkey; 5 Ankara University Faculty of Medicine, Department of Pediatric Endocrinology, Ankara, Turkey; 6 İstanbul University Faculty of Medicine, Department of Pediatric Endocrinology and Diabetes, İstanbul, Turkey; 7 Yüzüncü Yıl University Faculty of Medicine, Department of Pediatric Endocrinology, Van, Turkey; 8 Marmara University Faculty of Medicine, Department of Pediatric Endocrinology, İstanbul, Turkey; 9 İstanbul University Cerrahpaşa Faculty of Medicine, Department of Pediatric Endocrinology, İstanbul, Turkey; 10 Necmettin Erbakan University Faculty of Medicine, Department of Pediatric Endocrinology, Konya, Turkey; 11 Balıkesir Atatürk State Hospital, Clinic of Pediatric Endocrinology, Balıkesir, Turkey; 12 Gazi University Faculty of Medicine, Department of Pediatric Endocrinology, Ankara, Turkey; 13 İnönü University Faculty of Medicine, Department of Pediatric Endocrinology, Malatya, Turkey; 14 Osmangazi University Faculty of Medicine, Department of Pediatric Endocrinology and Diabetes, Eskişehir, Turkey; 15 Dokuz Eylül University Faculty of Medicine, Department of Pediatric Endocrinology, İzmir, Turkey; 16 Erciyes University Faculty of Medicine, Department of Pediatric Endocrinology, Kayseri, Turkey; 17 Çukurova University Faculty of Medicine, Department of Pediatric Endocrinology, Adana, Turkey; 18 Kanuni Sultan Süleyman Training and Research Hospital, Clinic of Pediatric Endocrinology, İstanbul, Turkey; 19 Dicle University Faculty of Medicine, Department of Pediatric Endocrinology, Diyarbakır, Turkey

**Keywords:** Pituitary, prolactin, children, microadenomas, macroadenomas, cabergoline, surgery

## Abstract

**Objective:**

We aimed to report the characteristics at admission, diagnosis, treatment, and follow-up of cases of pediatric hyperprolactinemia in a large multicenter study.

**Methods:**

We reviewed the records of 233 hyperprolactinemic patients, under 18 years of age, who were followed by different centers. The patients were divided as having microadenomas, macroadenomas, drug-induced hyperprolactinemia and idiopathic hyperprolactinemia. Complaints of the patients, their mode of treatment (medication and/or surgery) and outcomes were evaluated in detail.

**Results:**

The mean age of the patients with hyperprolactinemia was 14.5 years, and 88.4% were females. In terms of etiology, microadenomas were observed in 32.6%, macroadenomas in 27%, idiopathic hyperprolactinemia in 22.7% and drug-induced hyperprolactinemia in 6.4%. Other causes of hyperprolactinemia were defined in 11.3%. Common complaints in females (n=206) were sorted into menstrual irregularities, headaches, galactorrhea, primary or secondary amenorrhea and weight gain, whereas headache, gynecomastia, short stature and blurred vision were common in males (n=27). Median prolactin levels were 93.15 ng/mL, 241.8 ng/mL, 74.5 ng/mL, 93.2 ng/mL, and 69 ng/mL for microadenomas, macroadenomas, idiopathic hyperprolactinemia, drug-induced hyperprolactinemia, and other causes of hyperprolactinemia, respectively. Of 172 patients with hyperprolactinemia, 77.3% were treated with cabergoline and 13.4% with bromocriptine. 20.1% of the patients with pituitary adenomas underwent pituitary surgery.

**Conclusion:**

We present the largest cohort of children and adolescents with hyperprolactinemia in the literature to date. Hyperprolactinemia is more common in females and cabergoline is highly effective and practical to use in adolescents, due to its biweekly dosing. Indications for surgery in pediatric cases need to be revised.

**What is already known on this topic?**

Hyperprolactinemia affects gonadal function in the adolescent. Cabergoline is a useful treatment model for pituitary adenomas. Pituitary surgery for macroadenomas may be needed in some patients. Some drugs increase the prolactin level. Macroprolactinemia is one of the causes of hyperprolactinemia.

**What this study adds?**

Cabergoline is an effective treatment in the adolescent. There is no difference in terms of age between micro- and macroadenomas. Physicians should review the indications for surgery in macroadenomas. Macroprolactinemia is a neglected cause of hyperprolactinemia in cases with unexplained etiology.

## Introduction

Prolactin (PRL) is a luteotropic and pleiotropic hormone involved in many physiological functions, such as angiogenesis, the immune response, osmoregulation, reproductive behavior and lactogenesis. It is needed for the regulation of gonadal luteinizing hormone receptors in both genders, and it is necessary for lactation in females (1,2). Elevated PRL levels lead to various problems such as pubertal, mentrual and neurological. Stress, the use of drugs affecting the dopaminergic system and macroprolactinemia increase PRL to moderate levels, but pituitary adenomas increase PRL levels significantly (3,4,5). Signs and symptoms related to increased PRL such as oligomenorrhea, amenorrhea, and galactorrhea are more common in women in the adolescent period (4). In women, menstrual irregularity is the most common reason for referral, while males tend to experience intracranial pressure symptoms due to tumor growth. Medical treatment with a dopamine agonist is the first line treatment option for prolactinoma (5). Surgical intervention is considered in some cases (6).

Hyperprolactinemia is a common problem in adults and its etiology is different from that in children. Hyperprolactinemia is less frequently diagnosed in children. Accordingly, reports of pediatric hyperprolactinemia are less common.

In the present retrospective study, we aimed to investigate the differences between children and adults with hyperprolactinemia including etiology, treatment modality and treatment outcome in a large national cohort.

## Methods

### Patient Analysis

We reviewed 233 hyperprolactinemic patients under 18 years of age who were followed at 32 centers. Some of these cases have been reported previously (7,8,9). Hyperprolactinemia was diagnosed when repeated PRL concentrations were above 20 ng/mL. A microadenoma was defined as a pituitary tumor of less than 1 cm in diameter and a macroadenoma was defined as a tumor above 1 cm in diameter. The maximal diameter of the adenoma was evaluated using cranial magnetic resonance imaging (MRI). Drug-induced hyperprolactinemia was diagnosed if the patient had a history of medications such as antipsychotic, antidepressant or antidopaminergic agents. In this group PRL levels decreased to normal when the drug was withdrawn. If there was no mass evident on a pituitary MRI, no drug exposure and thyroid, kidney and liver dysfunction were excluded, the patient was accepted as a case of idiopathic hyperprolactinemia. Serum macroprolactin concentrations were sought in the group with idiopathic hyperprolactinemia and the complaints of these patients and their responses to treatment (medication and/or surgery) were evaluated in detail. The age, sex, and auxological evaluation results including height, weight and body mass index (BMI), and the respective standard deviation (SD) scores (SDS) of the patients were evaluated according the Turkish standards (10).

### Data Collection

This retrospective, multicenter, nationwide, web-based study was conducted using an electronic recording form (ERF) designed by two physicians (EE, OT) competent in PRL disorders as well as in ERF preparation. The ERF was used to collect the demographic data and clinical and laboratory findings of the patients with hyperprolactinemia. The ERF was uploaded to the CEDD Net Web Registry System website (www.cedd.saglik-network.org). Informed consent was obtained from the parents of the patients. The study protocol was approved by the Uludağ University Ethics Committee (number: 2015-19/10).

### Statistical Analysis

Statistical analyses were performed using SPSS v.23 for Windows (IBM Inc., Chicago, IL, USA). Normality was tested using the Shapiro-Wilk test. Data are presented as mean ± SD for parametric data and median (range) for non-parametric data. Student’s t-test was used for comparison of parametric variables, and Mann-Whitney U test was used for non-parametric data. Chi-square tests were used to determine significant differences in proportions among categorical variables. Spearman rank test was used for analysis of correlation among parameters. A p value of less than 0.05 was considered statistically significant.

## Results

**Figure 1:**
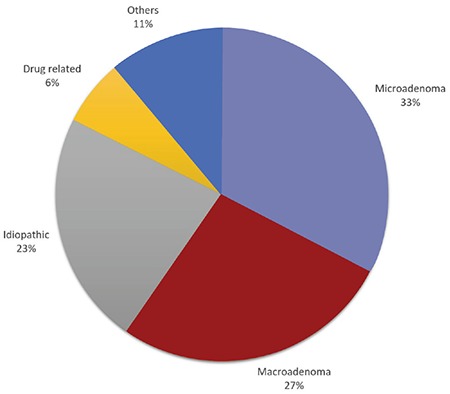
Diagnostic distribution of hyperprolactinemia patients

The median (range) age of the patients with hyperprolactinemia was 15.3 (0.12-17.7) years, and 88.4% (n=203) were females. In terms of etiology, pituitary microadenoma was observed in 32.6% (n=76), macroadenoma in 27% (n=63), idiopathic hyperprolactinemia in 22.7% (n=53) and drug-induced hyperprolactinemia in 6.4% (n=15) cases. Other causes of hyperprolactinemia were defined in 11.3% (n=26) (Table 1, Figure 1). Common complaints in females (n=206) were menstrual irregularity, headache, galactorrhea, primary or secondary amenorrhea and weight gain, whereas headache, gynecomastia, short stature and blurred vision were common in males (n=27) (Table 2). A family history of high PRL levels was detected in only seven cases. However mutation analysis of the *MEN* or *AIR* genes were not performed in that group. Patients with idiopathic hyperprolactinemia (n=53) complained of menstrual irregularity (49%), headache (22.6%), weight gain (22.6%), pubertal delay (20.7%) and galactorrhea (17%). Hyperprolactinemia was coincidentally detected in 13.2%. Other causes of hyperprolactinemia were sorted into non-pituitary masses (n=6), craniopharyngioma (n=5), macroprolactinemia (n=5), hypothyroidism (n=3), polycystic ovary syndrome (n=2), pituitary stalk interruption syndrome (PSIS) (n=2), rapid-onset obesity with hypothalamic dysfunction, hypoventilation, autonomic dysfunction syndrome (n=2) and tuberous sclerosis (n=1). In the group with drug-induced hyperprolactinemia, risperidone was used in nine of 15 (60%) cases and various antipsychotics or antidepressants were used in the other cases. Serum macroprolactin was measured in 48 cases with idiopathic hyperprolactinemia and detected in 5 (10.4%), with a median PRL level of 127 (63.5-200) ng/mL. The median PRL levels were 93.15 ng/mL, 241.8 ng/mL, 74.5 ng/mL, 93.2 ng/mL, and 69 ng/mL for microadenomas, macroadenomas, idiopathic hyperprolactinemia, drug-induced hyperprolactinemia and other causes of hyperprolactinemia, respectively (see Table 1).

**Figure 2:**
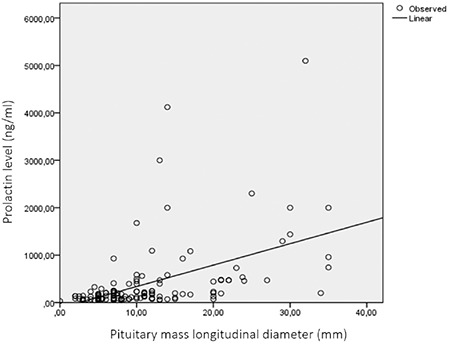
The correlation between serum prolactin levels and pituitary mass longitudinal diameter

When the cases with a prolactinoma (n=139) were divided into two groups, micro- and macroadenoma, there were no statistically significant differences in terms of age. In terms of gender distribution, 93.4% of microadenoma cases were female (71 female, 5 male) and 77.7% of macroadenoma cases were female (49 female, 14 male) (p<0.05). There was no significant difference in height, weight, height SDS, weight SDS, BMI, and BMI SDS between the two groups. However, BMI and BMI SDS tended to be greater in the macroadenoma group (Table 3). The maximal diameter of the adenomas was 5.9±2.1 mm in microadenomas and 17.3±7.4 mm in macroadenomas. There was a significant correlation between adenoma size and PRL level (p<0.05, r=0.494; see Figure 2).

Of 172 patients with hyperprolactinemia (micro- and macroadenomas plus some patients with idiopathic hyperprolactinemia), 77.3% were treated with cabergoline and 13.4% with bromocriptine. The remaining 9.3% were switched from bromocriptine to cabergoline because of treatment failure. The median (range) initial doses of cabergoline and bromocriptine were 0.5 (0.25-2.5) mg/week and 2.5 (0.5-7.5) mg/day, respectively, and the normalization period for PRL was 2 (0.5-47) months for cabergoline and 3 (1-17) months for bromocriptine, showing a statistically significant difference (p<0.170). There were no serious side effects for either drug. In total, 20.1% (28/139) of the patients with pituitary adenomas underwent pituitary surgery. Surgical option was the treatment of choice of the neurosurgeon or the patients who was not seen by an endocrinologist prior to consulting the surgeon. Transcranial surgery was performed in only two cases, while transsphenoidal surgery was performed in the remaining cases. In addition, 86.2% of these cases required a dopamine agonist after the operation. Only four cases received radiotherapy.

## Discussion

In this large, retrospective, multicenter cohort study, children and adolescents with hyperprolactinemia were evaluated and prolactinoma was detected in 60%. While some of the cases (23%) were idiopathic, others were due to various medications or other causes of hyperprolactinemia. Pituitary adenomas, most common in adults, are rare in children, and few studies have described the clinical signs and treatment outcomes of these adenomas in children. To date and to our knowledge, this is the largest cohort of pediatric patients with hyperprolactinemia in the pertinent literature. We therefore believe that this study will shed light on all aspects of this disease in the pediatric age group and reveal the differences from the adult population.

The ratio of macroadenoma appears to be very high in both girls (7%) and boys (28.5%) compared to adults. Adults are reported to have a higher prevalence of macroadenomas in males (11,12). A similar disproportion has also been reported in other pediatric cohorts (5,13).

The mean age of the two groups (micro-/macroadenomas) was not different significantly. This finding is contrary to the assumption that neglect of symptoms for many years leads to diagnosis of macroadenomas in men. The predominance of large tumors in men may be related to the biologic behavior of the prolactinomas.

Macroprolactinemia can be present in some cases with idiopathic PRL elevation. Macroprolactin is a big-PRL, accounting for 1% of total PRL (14). In some cases, this ratio increases and leads to a false diagnosis of hyperprolactinemia. Diagnosis is made with chromatography or polyethylene glycol analysis and there is no need for treatment. Macroprolactinemia is detected in 15-46% of hyperprolactinemic cases (14,15). In our study, macroprolactinemia was investigated in 48 cases and found in only five (10.5%). Some cases of idiopathic hyperprolactinemia are likely to have received unnecessary treatment because macroprolactinemia was not excluded. Macroprolactinemia should be considered in cases having non-specific symptoms and no abnormal features on pituitary imaging. Another cause of idiopathic hyperprolactinemia may be a PRL receptor mutation. Familial hyperprolactinemia has been described in some of these cases (14,16,17). In familial cases, *AIP* and *MEN1* should be included in the genetic analysis. In a study, patients with macroprolactinoma were found to have *AIP* (9%) and *MEN* (5%) variants, and dopamine agonist resistance was found in *MEN1* mutations (18). In our study, neither *MEN1* nor *AIP* were investigated.

Another cause of hyperprolactinemia is drug use. Many antipsychotic agents increase PRL by affecting the dopaminergic system (19,20). In total, 6% of our cases had increased PRL due to use of antipsychotic drugs. In this group the mean level of PRL was 100 ng/dL, with a maximum level of 200 ng/dL. The treatment of drug-induced hyperprolactinemia consists of a reduction of the drug dose or a transition to another drug. Pituitary imaging should be performed in cases with clinical symptoms. Other causes of hyperprolactinemia include craniopharyngioma affecting the pituitary gland, non-pituitary tumors and PSIS that may affect the tuberoinfundibular pathway and increase PRL.

Hyperprolactinemia is most frequently observed after the onset of puberty and in the female gender. Menstrual irregularity, galactorrhea and gynecomastia are commonly seen in these patients. In a study in which 27 pediatric cases were evaluated, 17 were female (63%) and had a mean age of 15.6 years (3). In our study, the mean age of the participants was 14.49 years, and the mean age of the patients with prolactinoma was 15 years.

In patients with macroprolactinoma, headache and visual problems are the first signs in males whereas primary or secondary amenorrhea is seen in all females (13). Oligomenorrhea and galactorrhea were the most common symptoms of macroadenoma in a study of 13 cases (10 female) (21). In another study, 80% of females with hyperprolactinemia presented with menstrual problems, galactorrhea and headache, while males presented with headache, visual problems and gynecomastia (18). In our study, 60% of girls had menstrual problems, 25% had headache and 25% had galactorrhea, whereas half of boys complained of headaches. To summarize, half of the males had headache, while half of the females presented with menstrual problems. It is possible that a larger pituitary adenoma could cause headaches due to delayed diagnosis. Other complaints in men were not specific. Hyperprolactinemia should be considered in the differential diagnosis of women with menstrual problems during puberty and cranial and pituitary imaging should be performed to elucidate the etiology.

In our series, interestingly, about 10% of cases experienced weight gain and 30.9% (n=72) of our cases were overweight or obese. The increase in BMI was encountered more frequently in macroprolactinoma. However, there was no correlation between BMI and PRL levels. In a study of 11 cases, six of whom were female, with hyperprolactinemia, who presented with short stature or growth deceleration, four had problems with weight gain and three had pubertal problems (4). In another study, 23% of the cases were referred to a physician with weight gain (18). In a study in which non-functional pituitary adenomas and prolactinoma were evaluated, BMI was reported to be significantly higher in the prolactinoma group. That group also had diminished growth hormone and insulin-like growth factor-1 levels (22). It has been shown that the modulatory effect of PRL may influence fat tissue and PRL changes body weight and composition. In 44 patients with prolactinoma, waist and hip circumference increased significantly, while fasting insulin and triglyceride were found to be elevated and fasting glucose and high-density lipoprotein cholesterol were normal (23). The relationship between PRL and obesity is unclear and remains to be elucidated. The PRL-releasing peptide (PrRP) is secreted by the hypothalamus, and it increases pituitary PRL production. It has also been found that PrRP is associated with nutrient and energy balance and that PrRP reduces weight gain and has an anorexigenic effect (24). The discovery of the relationship between PrRP and PRL could help in explaining weight gain in hyperprolactinemic individuals.

Dopamine agonists are first choice drugs to treat prolactinoma. Cabergoline has been used for many years as a highly effective and tolerable treatment. It was first used about 30 years ago in treating a patient who developed bromocriptine resistance (25,26). Cabergoline shrinks tumor cells and performs best with weekly dosing. It has been shown to be effective even in pituitary adenomas with no function (27) and has been used for years even in giant macroadenomas (28,29). In a study of 26 prolactinomas, bromocriptine was initiated in all cases, and conversion to quinagolide or cabergoline was made due to development of intolerance or resistance to bromocryptine (13). In our study, most of the centers preferred cabergoline as first line medication, and some had switched to cabergoline due to drug resistance. This study has shown that cabergoline can be used safely and effectively in children and adolescents.

Due to the high efficacy of dopamine agonists, surgery is rarely needed in prolactinomas. The rate of surgical treatment in our series was 20%, and this rate is likely to be higher than that reported in the literature on adult cases. Since adult studies generally show outcomes of cases who underwent surgery, it is not worthwhile to compare adult and child data for surgery ratio. In a pediatric study, seven of 27 patients (25.9%) were treated surgically, while 37.5% of adult patients with macroprolactinomas underwent surgery and 33% of these developed hypopituitarism (5,30). In another study evaluating nine surgically treated patients, transient complications, such as electrolyte disturbance, were observed postoperatively and no long-term sequelae were observed (31). Surgically, the transsphenoidal procedure was not associated with mortality, and no serious complications were observed (32).

It is unclear when surgical treatment of prolactinoma should be considered in children. It has been stated that transsphenoidal surgery can be used in patients who develop dopaminergic agonist intolerance or resistance or side effects from a drug. In addition, large adenomas causing visual problems and cerebrospinal fluid leakage due to pressure on the base of the skull are also candidates for surgery (33,34). In our cases, dopaminergic drugs had to be started or continued in 86.2% of the patients who underwent surgery. The surgical option should be avoided in children with prolactinoma because of recurrence after surgery and also because of development of various complications, including hypopituitarism, and the need to restart dopamine agonistic therapy.

### Study Limitations and Strength

The limitations of our study include the use of different methodology in laboratory data in different centers and also the use of different treatment modes and different approaches. *AIP* and *MEN* genes were not investigated. The strength of the study lies in its large case series, the inclusion of most pediatric endocrinology centers in the country and also its detailed data content.

## Conclusion

Hyperprolactinemia, which is more common in girls, is mostly caused by pituitary adenomas. Macroprolactinemia should be investigated in cases of unexplained hyperprolactinemia. Cabergoline is an effective treatment in children because of its weekly usage and the absence of significant side effects. The surgical option should not be considered in children, even in giant adenomas, because dopaminergic agonistic therapy is highly effective. Surgical indications need to be carefully considered by all relevant clinicians including endocrinologists and surgeons.

## Figures and Tables

**Table 1:**
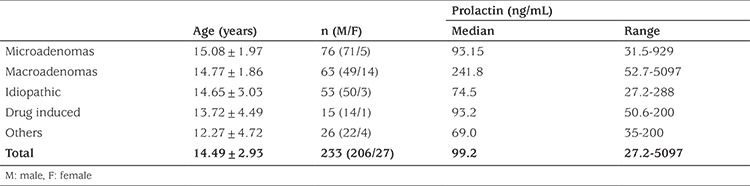
Age, gender distribution and serum prolactin level according to diagnosis

**Table 2:**
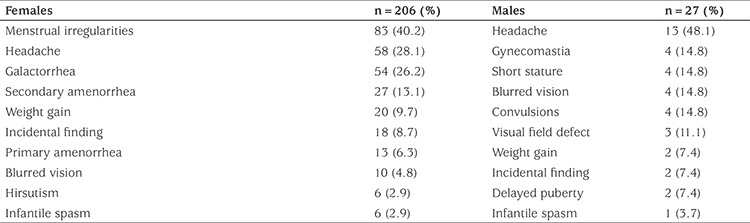
Common complaints and symptoms at diagnosis by gender in hyperprolactinemia patients

**Table 3:**
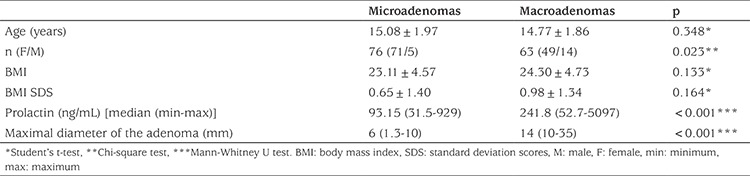
Age, gender, body mass index (BMI), BMI standard deviation scores, and pituitary adenoma diameter values in pituitary microadenoma and macroadenoma patients
